# Proximal major limb amputations – a retrospective analysis of 45 oncological cases

**DOI:** 10.1186/1477-7819-7-15

**Published:** 2009-02-09

**Authors:** Adrien Daigeler, Marcus Lehnhardt, Ammar Khadra, Joerg Hauser, Lars Steinstraesser, Stefan Langer, Ole Goertz, Hans-Ulrich Steinau

**Affiliations:** 1 Department of Plastic Surgery, Burn Center, Hand surgery, Sarcoma Reference Center, BG-University Hospital Bergmannsheil, Ruhr-University Bochum, Buerkle-de-la-Camp-Platz 1, 44789 Bochum, Germany

## Abstract

**Background:**

Proximal major limb amputations due to malignant tumors have become rare but are still a valuable treatment option in palliation and in some cases can even cure. The aim of this retrospective study was to analyse outcome in those patients, including the postoperative course, survival, pain, quality of life, and prosthesis usage.

**Methods:**

Data of 45 consecutive patients was acquired from patient's charts and contact to patients, and general practitioners. Patients with interscapulothoracic amputation (n = 14), shoulder disarticulation (n = 13), hemipelvectomy (n = 3) or hip disarticulation (n = 15) were included.

**Results:**

The rate of proximal major limb amputations in patients treated for sarcoma was 2.3% (37 out of 1597). Survival for all patients was 42.9% after one year and 12.7% after five years. Survival was significantly better in patients with complete tumor resections. Postoperative chemotherapy and radiation did not prolong survival. Eighteen percent of the patients with malignant disease developed local recurrence. In 44%, postoperative complications were observed. Different modalities of postoperative pain management and the site of the amputation had no significant influence on long-term pain assessment and quality of life. Eighty-seven percent suffered from phantom pain, 15.6% considered their quality of life worse than before the operation. Thirty-two percent of the patients who received a prosthesis used it regularly.

**Conclusion:**

Proximal major limb amputations severely interfere with patients' body function and are the last, albeit valuable, option within the treatment concept of extremity malignancies or severe infections. Besides short survival, high complication rates, and postoperative pain, patients' quality of life can be improved for the time they have remaining.

## Background

Due to sophisticated operative techniques and multimodal approaches, limb salvage in extremity malignancies has become possible in most of the cases [1,2]. Only advanced tumors, adjacent to crucial structures and close to the trunk, currently justify a sacrifice to an extremity. In those cases with excessive fungating tumor growth, ulceration, impending vascular disruption, intractable pain, paralysis, sensory disorders, lymphatic edema and a largely useless extremity, a proximal major amputation as a last resort may improve quality of live in an often palliative situation.

Against the background of rare detailed reports about long term outcome after proximal major amputations [3-10], that include parameters like pain, quality of life, and prosthesis usage, the aim of this study was to also focus on the clinical course, survival and its influencing factors, as well as the patients' satisfaction with the outcome after these disfiguring operations for malignant tumors.

## Patients and methods

From 1991 to 2006, 45 consecutive patients were treated by proximal major amputations at our institution. Patients who had received interscapulothoracic amputation (ISTA, n = 14), shoulder disarticulation (n = 13), hemipelvectomy (n = 3) or hip disarticulation (n = 15) were included in this retrospective study. Patients' data concerning their medical history and hospitalisation was obtained from the patients' charts. Follow-up data regarding the clinical course and outcome was collected from the patients' charts, interviews of the patients, their relatives and their general practitioners. Data concerning life quality and satisfaction with the aesthetic result was gathered at the regular follow-up visits at the time of complete wound closure and consisted of a simple score according to the German school mark system (1 = excellent, 6 = very poor). Postoperative ratings were compared to preoperative ratings that were asked for retrospectively.

At the time of treatment, the patients' ages averaged 56 years (range: 28–89 years); twenty (44%) were female. Eighty-two percent (n = 37) were operated on for sarcomas including two Ewing sarcomas, two chondrosarcomas, and one osteosarcoma, 18% (n = 8) for carcinoma (one breast, one Merkel cell, one metastasis of a pulmonary, and five squamous cell carcinomas). The rate of proximal major amputations in patients treated for sarcoma was 2.3% (37 out of 1597). Nineteen of the 45 patients with malignant disease (42%) were operated on for recurrent tumors. Twenty-eight out of those 45 had metastatic disease at the time of the proximal major amputation (62%) and were treated with palliative intention, 31 had already received chemotherapy and radiation, respectively (69%), of whom 19 had undergone both treatment modalities. Thirty-eight of the patients had an average of 4.2 previous diagnosis-related operations (range: 1–15), with up to nine operations for local recurrences.

**Figure 1:**
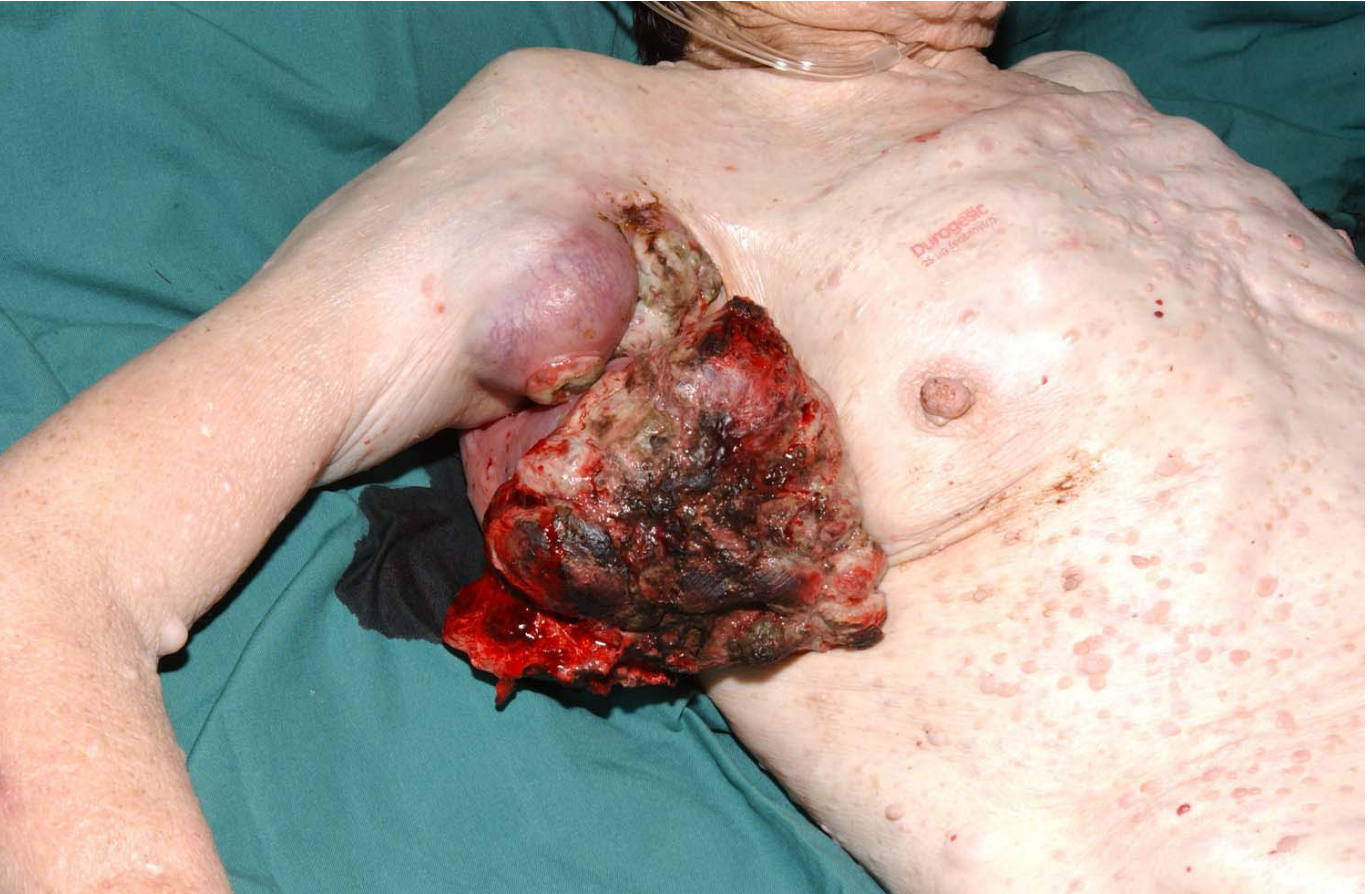
**Preoperative aspect of a patient with the forth recurrence of a malignant periperal nerve sheath tumor (MPNST) and a history of neurofibromatosis Recklinghausen resulting in interscapulothoracic amputation**.

**Figure 2:**
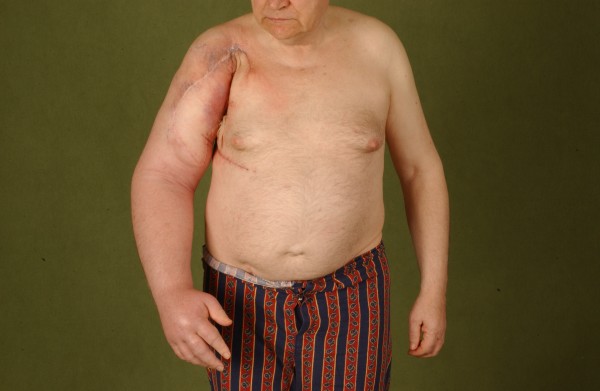
**Preoperative aspect of a patient with recurrent MFH/NOS after limb sparing resection and reconstruction with pedicled latissimus dorsi flap showing a massive lymphatic edema**.

**Figure 3:**
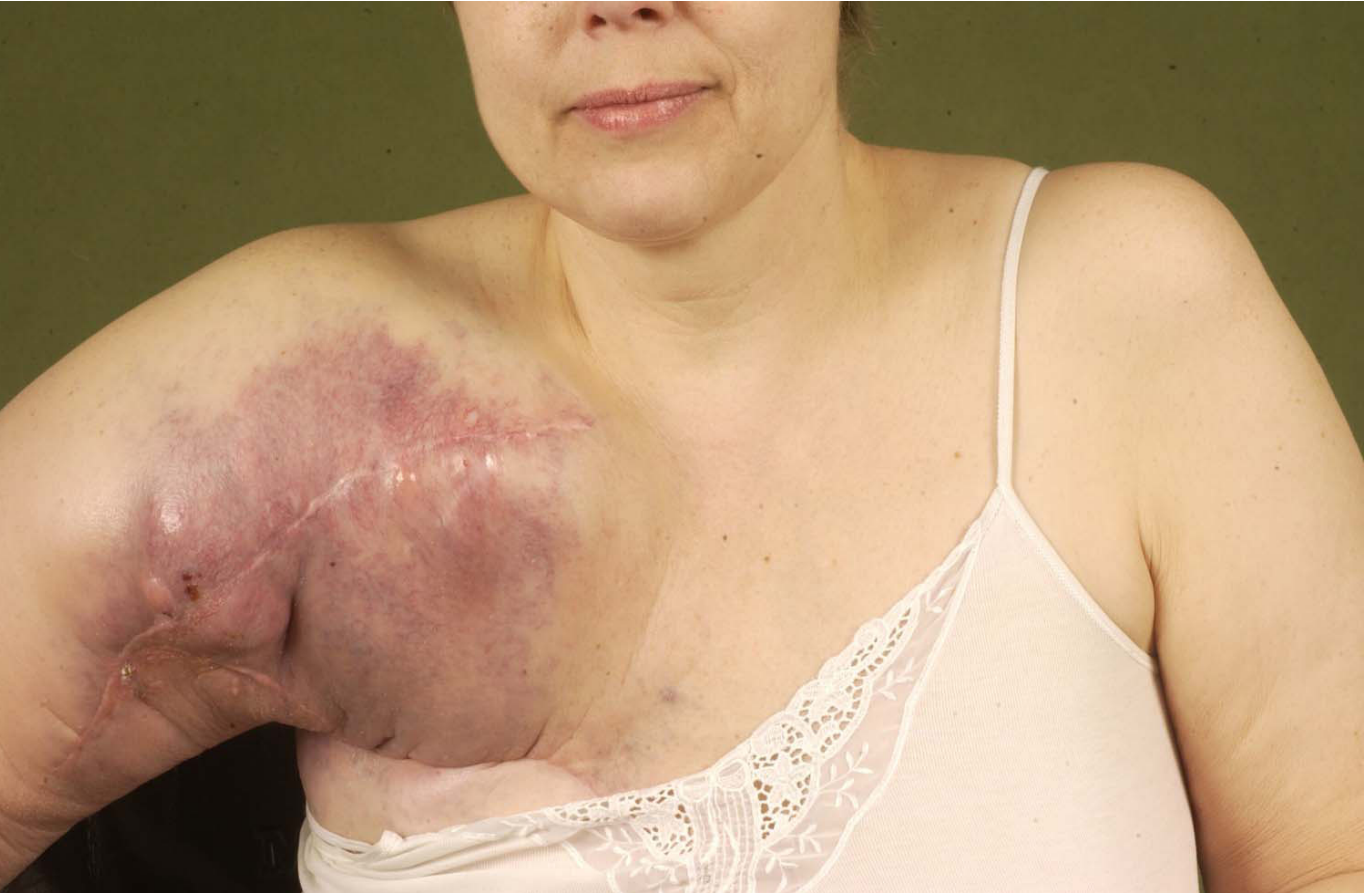
**Aspect of recurrent soft tissue sarcoma in the right axillary region**.

**Figure 4:**
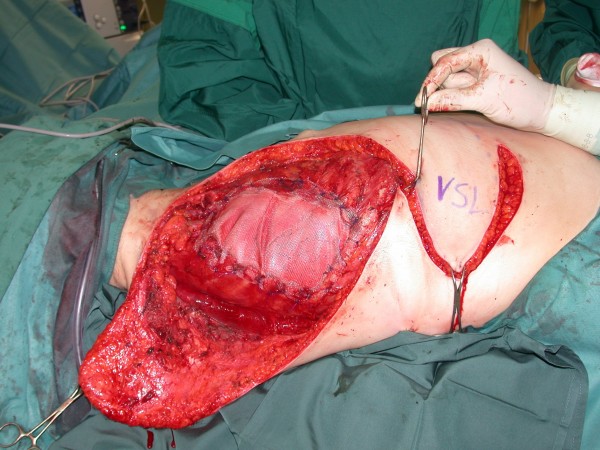
**Aspect after tumor resection including parts of the thoracic wall and reconstruction with synthetic mesh. Large fasciocutaneous flaps are prepared to cover the defect**.

**Figure 5:**
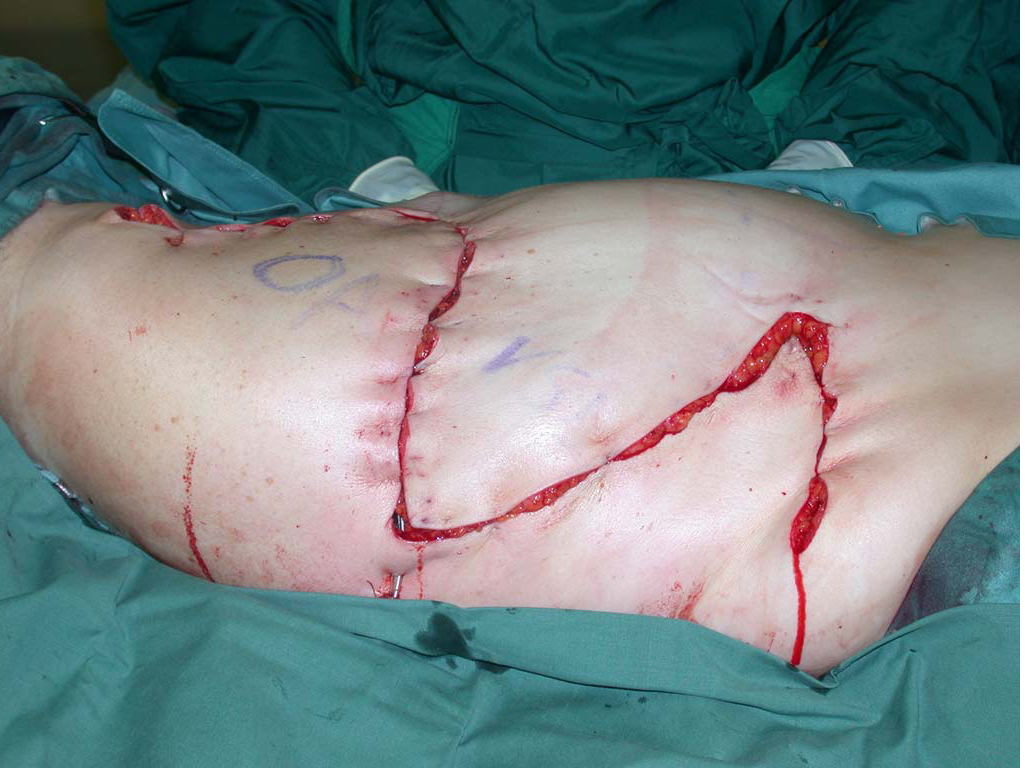
**Postoperative aspect after positioning of the flaps with sufficient coverage of the defect**.

Several preoperative aspects are illustrated in figures 1 and 2. Figures 3, 4 and 5 illustrate the preoperative and intraoperative aspects of an ISTA including thoracic wall resection and reconstruction.

Three patients were lost to follow-up and no follow-up information could be gained; 38 patients (84%) had already died. The mean follow-up time was 20 months (range 0–118 months). Four patients who were alive could be interviewed personally. In the other cases, data was obtained from the close relatives and the attending general practitioners. Data was acquired retrospectivelly and may therefore be biased.

For statistic analysis, SPSS Version 15.0 for Windows (SPSS Inc., Chicago, USA) was used. The correlation of different events was calculated by crosstabs (chi-square, Pearson): prosthesis usage, quality of life, pain, complications; the means of parametric data by ANOVA (prosthesis usage) and the rank of nonparametric data by Mann-Whitney, Wilcoxon and Kruskal-Wallis tests (Quality of life, pain).

P-values ≤ 0.05 were considered significant. Survival and influencing factors were calculated using the Kaplan-Meier Method (Log-Rank (Mantel-Cox)).

This study was approved by the Ethics Committee of the BG-University Hospital Bergmannsheil (number: 3041-07).

## Results

In 17 patients who had no metastases at the time of the amputation, complete resection (R0-status) could be achieved in 11 (65% of 17). In 4 patients (24% of 17) positive surgical margins (R1-status) were histologically documented and in 2 (12% off 17) additional patients macroscopic tumor residuals were left in situ (R2). Seven (out of the 28 patients with metastases were resected with clean surgical margins, 11 had positive margins, and in 10 cases macroscopic tumor residuals remained in situ.

In most cases, local muscle and fasciocutaneous flaps were sufficient to close the defects. After hemipelvectomies and hip disarticulations dorsal flaps were used to cover the defects; an Epaulette flap was used in only three cases as previously described in patients with ISTA [11]. No intra-operative death occurred but one (2%) patient died within the 30-day postoperative period after hemipelvectomy for recurrent and ulcerated soft tissue sarcoma three days later, due to septic multi organ failure (MOF). Further information about the intra- and postoperative course of the patients is given in table 1.

**Table 1:** Intraoperative usage of erythrocyte concentrates and prosthetic mesh; length of stay (LOS) in the hospital and length of treatment at the intensive care unit (ICU) in days.

	HD	HP	SD	ISTA
EC	4 (0–10)	13 (3–25)	2 (0–4)	3 (0–6)

Prosthetic mesh	2	2	0	12

LOS	30 (11–65)	17 (7–28)	12 (5–23)	25 (12–47)

ICU	8 (0–59)	8 (2–20)	1 (0–3)	5 (2–27)

HD = hip dysarticulation, HP = hemipelvectomy, SD = shoulder dysarticulation, ISTA = interscapulothoracic amputation, EC = erythrocyte concentrates.

Postoperative complications were observed in 20 patients (44%). Sixteen patients had to be re-operated on those complications (average 2 times, range: 1–6 times). Among the complications, partial flap necrosis was the most common (n = 9, 20%), followed by wound infection (n = 7, 16%), hematoma, seroma and wound dehiscence in two cases each (4%) and one hernia after hip disarticulation. The occurrence of wound healing difficulties such as wound infection, seroma, and necrosis was higher in patients with preoperative radiation of the amputation area but the finding was not significant (p = 0.153). Preoperative chemotherapy (p = 0.890) and the amount of intra-operative blood transfusion (p = 0.874) also had no significant influence on the occurrence of wound healing difficulties.

In spite of the radical operation, local recurrence was observed in 8 tumor patients (18%) of whom two had received a complete resection (R0), four had been resected with positive surgical margins (R1) and two had only received a tumor mass reduction (R2). After the amputation operation these patients had a progression free interval of 7 months on average (range 1–20 months) and died after 17 months (range 4–39 months).

**Figure 6:**
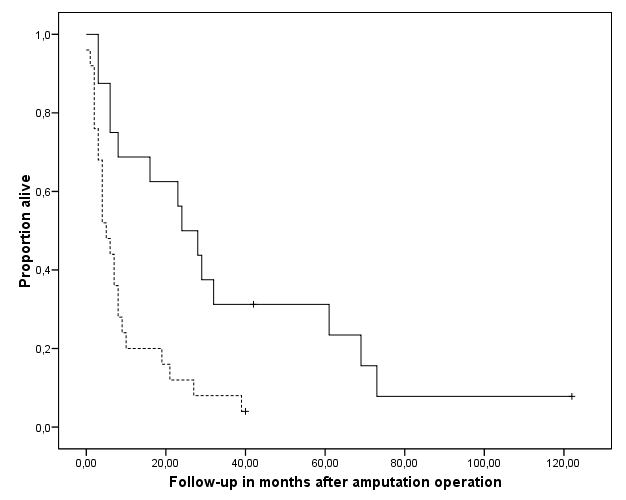
**Overall survival after the amputation operation with free surgical margins (n = 16, continuous line) and with positive surgical margins (n = 25, broken line)**. The tick marks indicate last follow-up. The difference was significant (Kaplan Meier Log-Rank, p = 0.002).

**Figure 7:**
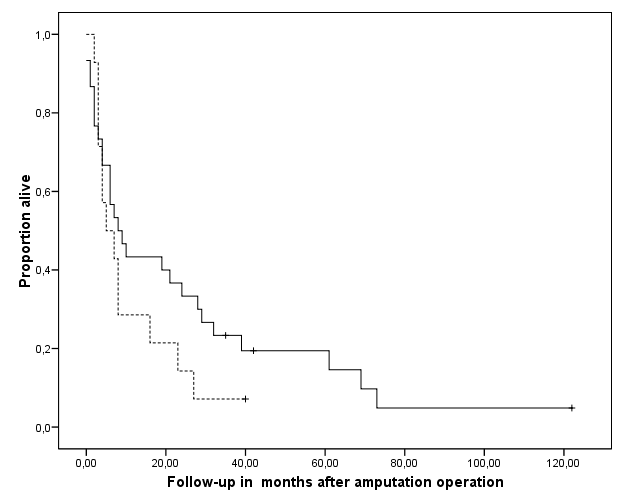
**Overall survival after amputation of patients who received chemotherapy (n = 14, broken line) and who did not (n = 30, continuous line)**. No significant difference could be detected (Kaplan Meier Log-Rank, p = 0.299). The tick marks indicate last follow-up.

**Figure 8:**
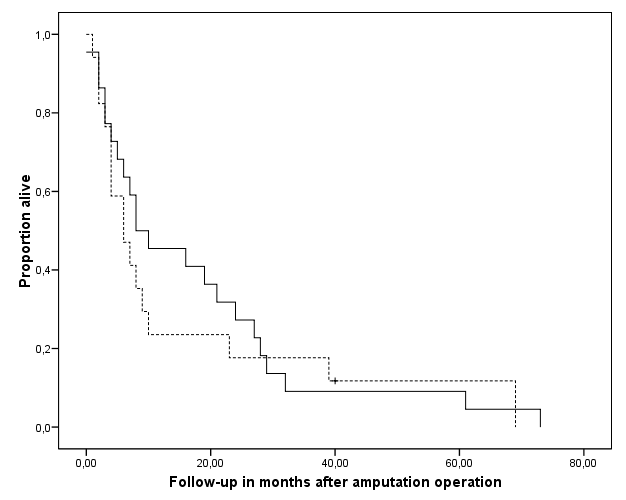
**Overall survival after amputation of patients who received adjuvant radiation (n = 17, broken line) and who did not (n = 22, continuous line)**. The differences were not significant (Kaplan Meier Log-Rank, p = 0.631). The tick marks indicate last follow-up.

**Figure 9:**
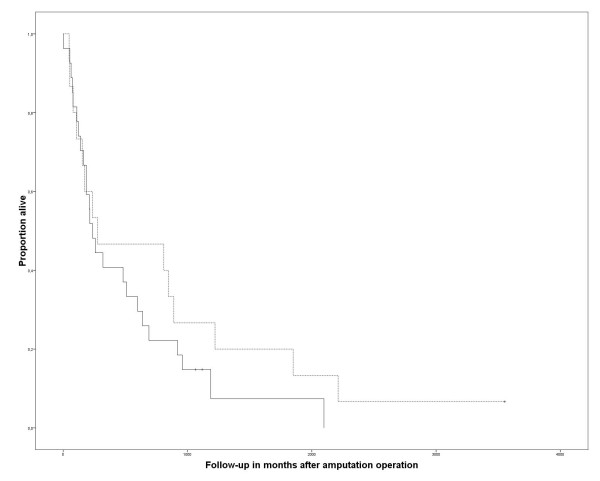
**Overall survival of patients treated in curative intention (n = 17, continuous line) and palliative intention (n = 28, broken line)**. The differences were not significant (Kaplan Meier Log-Rank, p = 0.307). The tick marks indicate last follow-up.

Survival for all patients was 42.9% after one year and 12.7% after five years. The four patients who were alive could be interviewed personally at follow-up times of 3, 40, 42, and 118 months. Survival was significantly reduced in patients with positive surgical margins (p = 0,002) (Fig 6). Neither the primary diagnosis that led to the amputation, nor adjuvant chemotherapy (chi-square 1.447, p = 0.229) (Fig 7) or radiation (chi-square 0.230, p = 0.631) (Fig 8) after the amputation had significant influence. Patients who were operated on in palliative intention lived shorter than those treated with curative intention (Fig 9), but the difference was not significant (chi-square 1.042, p = 0.307).

For postoperative pain relief, including pain at the operation site as well as phantom pain, 24 patients received opioids, 12 pain catheters, and ten received other pain medication. Pain intensity as well as the occurrence of phantom pain was documented in 40 and 39 patients, respectively. All except one suffered from pain in the operated region. Pain intensity or the occurrence of phantom pain was not significantly influenced by the fact if postoperative pain therapy was performed by i.v. or oral opioids, catheters or other modalities of pain management (p = 0.512) and by the kind of amputation that was performed (p = 0.315). Pain reduction could be achieved in 46% of the patients in comparison to the preoperative situation (when receiving the same medication as preoperative), whereas 31% had more pain afterwards and 22% reported no change. Neither the different preoperative diagnoses (p = 0.702), nor the kind of amputation operation (p = 0.512), had significant influence on the changes from pre to postoperative pain.

Twenty-four patients received a custom made prosthesis but only seven out of 22 of those that could be followed up used it regularly (32%). Eleven used it only rarely (50%) and four had never used it (18%). The reasons given for rare or no use of the prosthesis were complicated handling and inoperability in all cases. One patient could not wear it because it caused intolerable pain (table 2). Statistical analysis could not detect a parameter (age, gender, preoperative diagnosis, kind of amputation) that had significant influence on the fact if the prosthesis was used or not.

**Table 2:** Usage of the custom made prosthesis by the patients.

	HD	HP	SD	ISTA
N	15	3	13	14

N with custom made prosthesis	8	0	6	9

Regular use	3	0	2	2

Rare use	3	0	4	5

No use	2	0	0	2

HD = hip dysarticulation, HP = hemipelvectomy, SD = shoulder dysarticulation, ISTA = interscapulothoracic amputation.

For 39 patients, a subjective rating of the cosmetic result could be obtained. The average rating was 3.4 (1 = excellent, 6 = very poor) with a range from 2 to 5, whereas the average rating in the hemipelvetomy cases was 3.0, in the hip disarticulation group was 3.2, in the ISTA patients was 3.2 and 3.3 was the rating in the shoulder disarticulation group. In 32 patients an assessment of their quality of life changes after the operation was documented: Six-teen considered it improved postoperatively, 11 felt no change, and five found it worse than before. The improvement in the hip disarticulation group was significant (p = 0.029), the shoulder disarticulation group tended to do better (p = 0.052), but the changes in the ISTA-patients were not significant (p = 0.584). Statistical analysis in the hemipelvectomy group could not be performed because quality of life data was documented in two patients only. Quality of life after wound healing was independent from the localization of the amputation (p = 0.624), postoperative pain management (p = 0.563), and the occurrence of postoperative complications (p = 0.410). The higher the preoperative pain intensity (p = 0.009) and the poorer the quality of life (p = 0.001), the better was the postoperative quality of life. No other factor, be it the preoperative diagnosis or the kind of amputation, contributed significantly to changes in quality of life.

In 37 patients, we documented whether patients would undergo the procedure again.

Twenty-two had answered yes (59%), 15 (41%)would not agree to the procedure again with their present knowledge.

## Discussion

New surgical techniques and a better understanding of the tumor biology, supported by multimodal therapy, presently make functional limb sparing tumor resections the therapy of choice and have reduced proximal major limb amputations to rare indications [1,12,13]. The rate of 2.3% of all patients treated for sarcoma at our institution further confirms this statement. While microsurgery offers a wide range of reconstructional options [11,14,15] after those amputations, most of our cases who were treated in palliative intention, did not qualify for sophisticated procedures. Except three Epaulette flaps, sufficient closure could be achieved with local flaps as also suggested by other authors [16-18].

Intra-operative blood replacement largely concurs with the volumes reported in the literature [19,20]. The length of stay largely corresponded to the extent of the operation. Interestingly, patients with ISTA stayed approximately as long as those with hemipelvectomy or hip disarticulation. This may be caused by the fact that many of them had received additional thoracic wall reconstruction or by the longer time the patients needed to adapt to loss of the limb.

The results of this study show that complete resection correlated with longer survival. The probably aggressive tumor biology and incomplete resection may explain the high local recurrence rate [1]. So far it has not been shown if incomplete resection is only correlated to or the reason for worse survival. The fact that complete resection of the lesion contributed significantly to improved survival is quite comprehensible considering that patients with localized disease were resected in curative intention and therefore more aggressively. In the other cases, the amputations were operations of desperation employed when all other methods had failed. Patients who presented with recurrent tumors did worse perhaps because distant and eventually occult metastases were already prevalent in many cases at time of amputation [21]. Interestingly the survival of patients who did not present metastatic disease at the time of the amputation was not significantly longer than in patients with disseminated disease. Most of the patients initially treated with curative intention may already have had occult metastasis.

The negative patients selection with a predominance of patients with recurrent and metastatic disease is responsible for the low long term survival rates that corresponds to time frames given by other authors after proximal major amputations for sarcoma, who reported five-year survival rates of 10–39.3% [19,21-24]. In a series of palliative forequarter amputations, the patients' post-operative survival ranged from 3 to 12 months [25]. In our series, radiation therapy and postoperative chemotherapy could not improve survival. This is probably due to the aggressive tumor behaviour and the fact that radiation can improve local control at best but not help in a case of disseminated disease that was already present in the majority of our patients. Furthermore, patients with progressed disease are regularly treated more often with chemotherapy or radiation than those with localized lesions [26-29], contributing to the bias. In this context it should be mentioned that the low number and the heterogeneous population of patients and the retrospective study design do not allow for meaningful conclusions.

The patients' benefit of proximal major amputation can be questioned against the background of those numbers, but pain relief and the improvement of quality of life quantified in this study may suggest otherwise.

In contrast to other series, we could not detect any significant influence of the postoperative pain management on long term pain sensation or the occurrence of phantom limb syndrome in our patients, of whom the vast majority suffered from this complication [30]. Phantom limb syndrome was reported by other authors to develop in up to 86% after upper limb and 82% after lower limb amputation. In addition to other factors, this may become such a burden for the patients concerned that up to up to 32% of the amputees harbour suicidal ideas and 65% suffer from sadness [31,32]. Many authors demand that sufficient pain management has to start prior to surgery to avoid pain memories in phantom limbs; spinal or plexus anaesthesia may further reduce the risk [33]. Injection of anaesthetic into the severed nerve ends may provide some long term pain relief and reduce the risk of phantom pain [34,35].

A study dealing with long term pain results showed, that pain decreases slightly with time, with 72% having phantom pain after 8 days, 65% after 6 months, and 60% two years postoperative [36], but, in the long run, only seven percent of the patients could substantially be helped by the more than 50 types of therapy [37].

**Figure 10:**
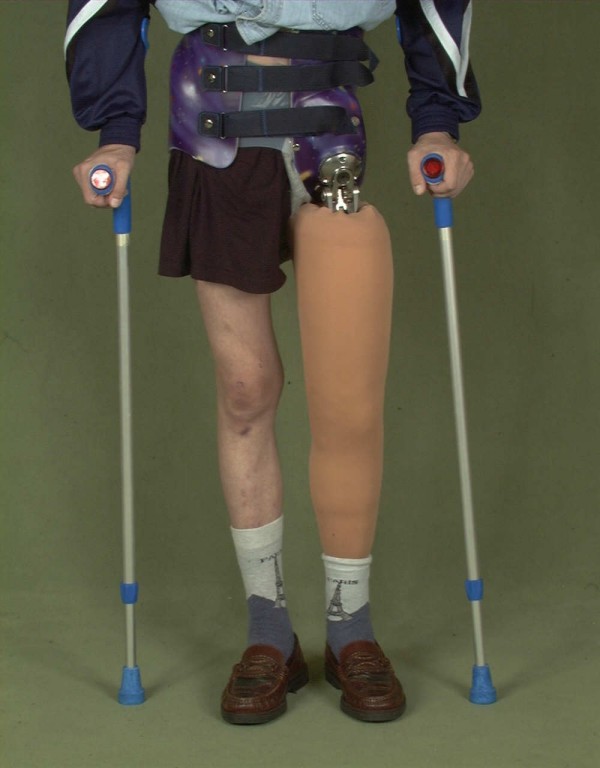
**Custom made prosthesis after hip disarticulation**.

Our findings of only moderate acceptance of the prosthetic supply comply with the reports of others who stated that amputations at trunk level lead to a three times higher prosthesis rejection rate than distal amputations [38] and documented 57% prosthesis rejection rates e.g. after ISTA [32]. The most named reason was lack of function and complicated handling. Considering that a prosthesis after hemipelvectomy weighs at least 5 kg and that patients (if ever getting walking on crutches again after hip disarticulation and hemipelvectomy) have an increased energy expenditure of 100 to beyond 200% [18,39-41] (Fig 10), the high rejection rates are quite comprehensible. Especially obese patients are at risk for a wheel chair live after amputation of the lower extremity. On the other hand, in case of loss of the trochanter and the tuber ischiadicum, sitting becomes almost impossible without prosthetic fitting because of the reduced sitting surface. The high cost for leg prosthesis (about 4,000€–16,000€) [24] calls into question the sense of general prosthetic supply for every patient. Even modern myoelectric prosthetic devices can not sufficiently replace arm and hand function. Therefore, an adequate aesthetic substitute like a simple shoulder pad that improves the fitting of clothes by correcting the body contour may be preferred by ISTA or shoulder dysarticulation patients.

We could not identify predictive factors of long term use of the prosthesis to justify an exclusion of patients from prosthetic fitting. Therefore prosthesis initially should be provided for every patient who asks for it, but the possible benefit and disadvantages should be discussed in detail.

**Figure 11:**
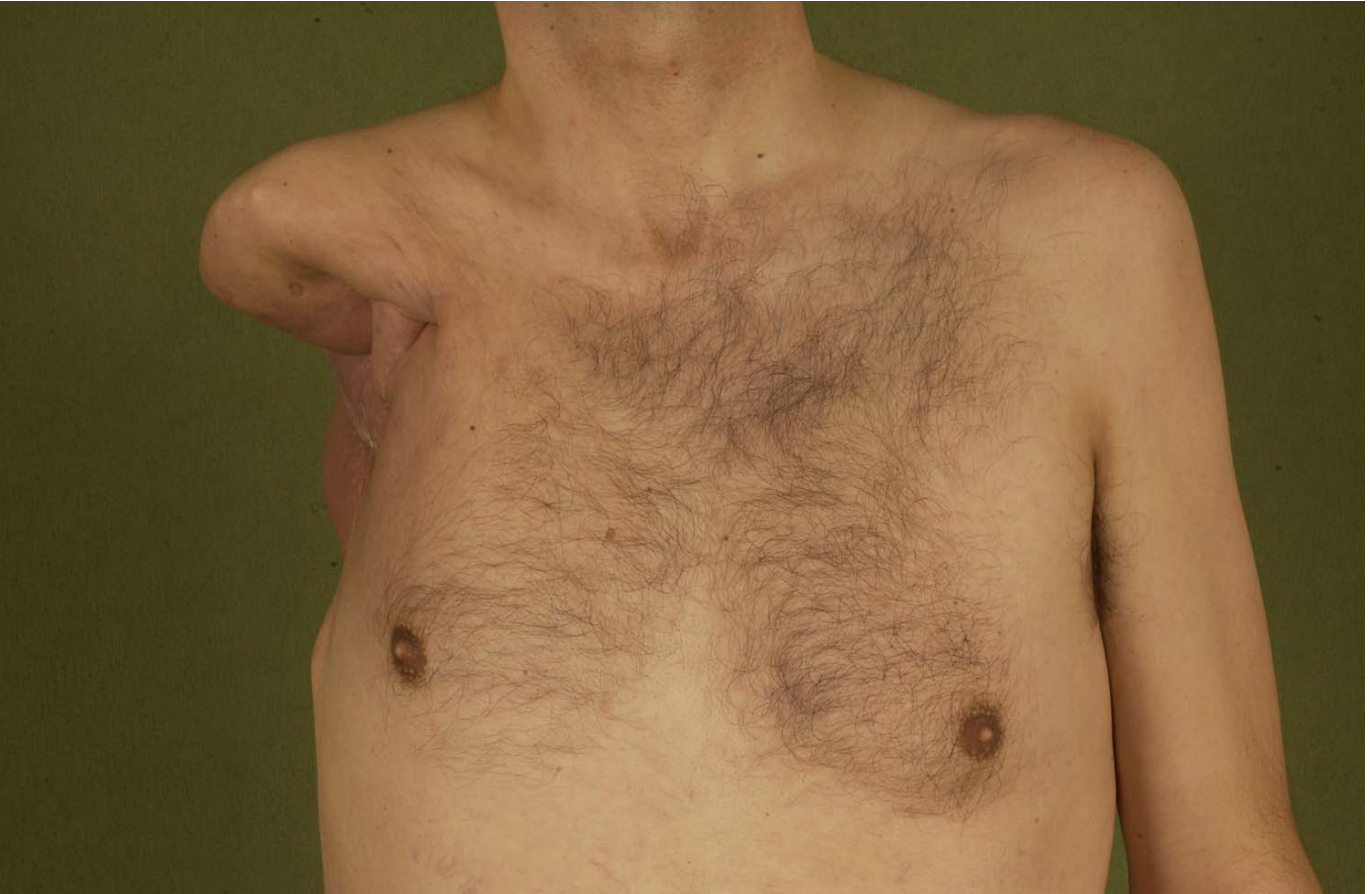
**Postoperative aspect after extended shoulder disarticulation for synovial sarcoma**.

A limb is an essential part of the patients' body schema and the often disfiguring postoperative results put strain on the patients (Fig 11). Additionally the enormous operative and postoperative effort and the costs accompanied by rare cases of cure and usually short survival may make proximal major limb amputations a questionable procedure. On the other hand reports about fully reintegrated persons as well as pregnancies and successful uncomplicated deliveries in females after hemipelvectomy show that acceptance can be achieved in patients with a positive attitude [42,43].

Most of the data was acquired retrospectively limiting the interpretation of the results, but in agreement with other studies that reported pain relief and improvement of quality of life [25] we affirm that proximal major amputations are still a valuable treatment option in selected cases with excessive fungating tumor growth, ulceration, impending vascular disruption, intractable pain, paralysis, sensory disorders, lymphatic edema or a largely useless extremity. The majority of our patients accepted the aesthetic outcome and most of them felt a significant improvement of quality of life after the operation. Our data show that especially those patients with low preoperative life quality and high pain levels benefitted most from the amputation operation.

## Competing interests

The authors declare that they have no competing interests.

## Authors' contributions

ML participated in the study design and helped drafting the manuscript. AK acquired the data and did the statistical analysis. JH prepared the figures and did the literature research. LS helped aquiring the data and corrected the manuscript. SL was helpful conceptualizing the study and weighed the data. OG interpreted the data and was helpful with the review of the literature. HS initiated the study and corrected the manuscript. All authors read and approved the manuscript.
